# Corrigendum: Deep Learning for Whole Slide Image Analysis: An Overview

**DOI:** 10.3389/fmed.2020.00419

**Published:** 2020-08-19

**Authors:** Neofytos Dimitriou, Ognjen Arandjelović, Peter D. Caie

**Affiliations:** ^1^School of Computer Science, University of St Andrews, St Andrews, United Kingdom; ^2^School of Medicine, University of St Andrews, St Andrews, United Kingdom

**Keywords:** digital pathology, computer vision, oncology, cancer, machine learning, personalized pathology, image analysis

Equal contribution was incorrectly attributed to the authors. Instead, the statement “These authors have contributed equally to this work” should be removed.

In the published article, Peter D Caie was incorrectly listed as the corresponding author. Instead, Neofytos Dimitriou should be the corresponding author. The corresponding author's email address should read neofytosd@gmail.com.

The authors apologize for these errors and state that these do not change the scientific conclusions of the article in any way. The original article has been updated.

